# ‘You get a different mindset’ – primary care physicians’ perceptions of interprofessional medication reviews for patients living independently

**DOI:** 10.1080/02813432.2025.2604036

**Published:** 2026-01-02

**Authors:** Annika Dobszai, Sara Modig, Cecilia Lenander, Beata Borgström Bolmsjö

**Affiliations:** ^a^ Center for Primary Health Care Research, Department of Clinical Sciences, Malmö, Lund University, Malmö, Sweden; ^b^ University Clinic Primary Care Skåne, Lund, Sweden; ^c^ Department of Medicines Management and Informatics in Skåne County, Malmö, Sweden; ^d^ Clinical Memory Research Unit, Department of Clinical Sciences Malmö, Lund University, Lund, Sweden

**Keywords:** Independent living, medication review, pharmacists, physicians, primary care, qualitative research

## Abstract

**Objective:**

In primary health care in southern Sweden, interprofessional medication reviews are conducted, to a limited extent, for patients living independently, with the purpose of optimising their pharmacological treatment. These medication reviews have identified and addressed many important issues regarding the patients’ medication. However, there is limited knowledge regarding the participating physicians’ perspectives on the work. Gaining a better understanding of the physicians’ perspectives could contribute to the optimisation of the interprofessional medication review model used in primary health care. This study aimed to explore physicians’ perceptions and experiences of interprofessional medication reviews in primary health care for patients who live independently.

**Methods:**

We conducted a qualitative study based on semi-structured discussions in four focus groups with a total of 24 participating primary care physicians. The discussions were transcribed verbatim, condensed, coded and categorised using content analysis.

**Results:**

Through our analysis, we identified four categories: *obstacles and facilitators, interprofessional collaboration with the pharmacist*, *the physician’s responsibility* and *value-adding aspects*. An underlying theme, *a value-creating intervention with logistical barriers,* emerged from the latent material.

**Conclusion:**

Physicians expressed a consistently positive view of medication reviews for independently living patients, highlighting their value for patient safety, clinical decision-making, and learning. However, practical barriers, such as time constraints and unclear routines, were seen as key obstacles to implementation. These findings reflect the dual nature of medication reviews as a value-creating intervention with logistical challenges, which need to be addressed to support broader integration in primary care.

## Introduction

All pharmacological treatments require regular systematic evaluation and medication reviews (MRs) can be a method to accomplish this. MRs are performed in various regions across the world with differing structures and in a variety of settings, with the purpose of preventing and solving medication-related problems. The WHO suggests MRs to address inappropriate medications and polypharmacy, and as a component of a personalised care plan [1]. Internationally, a range of models for MRs exists, differing in structure, setting and professional roles. In Australia, accredited pharmacists conduct home-based reviews and provide recommendations to general practitioners, as part of their Home Medicines Review program [2]. Structured MRs in the United Kingdom are integrated into care teams, with pharmacists actively participating in deprescribing and medication optimisation [3]. The model in the United States often includes MRs as a part of broader pharmacist-led services within primary care. Under Collaborative Practice Agreements, pharmacists may initiate, modify, or discontinue treatments in collaboration with physicians [4]. These examples reflect the variation in how MRs are organised across different healthcare systems.

In Sweden, MRs are mandated by the Board of Health and Welfare at different levels [5]. For many years, interprofessional teams in Skåne County in the south of Sweden have been working with structured MRs with the aim to optimise the individual patient’s medication treatment in case of, for example, polypharmacy or extensive co-morbidity [6–9]. The work has mainly concerned patients living in institutional settings or patients in hospitals. In recent years, a model for interprofessional MRs in Skåne has been developed in primary care for patients living independently [10,11]. A symptom assessment scale, PHASE-20 (PHArmacotherapeutical Symptom Evaluation, 20 questions) [12] is answered by the patient. PHASE-20 is a validated tool, consisting of questions for the patient to identify symptoms of, for example, adverse drug reactions. A clinical pharmacist evaluates PHASE-20 in light of the current medication list and a review of the patient’s medical record, identifying potential drug-related problems. The pharmacist compiles any identified drug-related problems in the electronic medical record, together with suggested measures. The pharmacist and the physician then have either a written contract, or a combination of written contract and verbal discussion regarding the patient’s medication. Subsequently, the physician decides on appropriate measures and follow-up actions based, among other factors, on the pharmacist’s summary.

Although the model is available to all physicians in public primary care, only a limited number of MRs are conducted for patients living independently. Previous studies have explored physicians’ experiences with MRs in other healthcare systems and settings. Dhillon et al. found that Australian general practitioners appreciated pharmacist input but noted workflow challenges [13] and in UK, Duncan et al. reported that physicians valued collaboration with pharmacists but emphasised the need for clear role definitions [14]. However, the specific model for interprofessional MRs in Skåne for this patient group has not previously been investigated.

The model is used to some extent for patients living independently, but MRs in primary care are still mainly conducted for patients in institutional settings. In order to identify potential needs for modification of the model and for further implementation, the physicians’ perceptions and experiences of the work were sought in this study.

## Methods

### Design

In this qualitative study, focus group discussions were performed to explore primary care physicians’ perceptions and experiences of interprofessional medication reviews (MRs) in primary healthcare for patients living independently. The physicians’ thoughts about pharmaceutical support in clinical work from a wider perspective were also included.

### Setting and participants

Physicians working in public primary healthcare centres, where MRs for patients living independently had been conducted, were purposively selected for the focus group discussions. Both physicians with experience of MRs and those without experience were included to get different perspectives and yield rich material. Each group consisted of three to nine physicians. The participants were united by common experiences as primary care physicians, where interaction between them could generate a good breadth in the answers. This was considered desirable as the implementation of MRs in this setting has not previously been studied.

Six healthcare centres were contacted by email, but one declined to participate and one was unable to allocate time when the meeting was scheduled. After three focus groups, the research team observed a saturation point, as no new information emerged during the third session. However, it was considered that additional insights might exist among physicians with greater experience in conducting MRs for patients living independently. Therefore, the fourth group was modified to consist solely of such physicians, in order to explore these perspectives more deeply. After obtaining information from the more experienced physicians in the fourth focus group, saturation was deemed to have been reached with respect to the study’s research questions.

Overall, the focus groups consisted of a mix of participants in terms of gender, experience of MRs and work experience, as presented in Table 1. The focus group discussions were performed between October 2023 and January 2024. A semi-structured interview guide with open-ended questions was used in all focus groups (see Appendix A). The guide was developed collaboratively within the research team and refined through discussion, although it was not pilot tested prior to data collection. Each focus group discussion lasted approximately just under one hour. Some field notes were made. One member of the research team (AD) acted as moderator, and two others (BBB and SM) alternated as co-moderators. Three of the discussions took place at the health centres where the physicians worked. The fourth was held as a digital meeting using the Teams platform.

**Table 1.** Characteristics of the participants.

	Group 1 *n* = 5	Group 2 *n* = 9	Group 3 *n* = 7	Group 4 *n* = 3	Total *n* = 24
Meeting format	face to face	face to face	face to face	digital	
Female/Male	3/2	5/4	4/3	2/1	14/10
General Practitioner/Resident physician in family medicine/Licensed physician	3/2/0	5/3/1	4/3/0	3/0/0	15/8/1
Experienced medication review(s) for patients in ordinary living (yes/no)	5/0	3/6	3/4	3/0	14/10

Before the focus group sessions, written information was sent to the participants with information about the project, the purpose of the interview and some points to reflect on (see Appendix B). Informed consent was obtained from all included participants and ethical approval was granted (Dnr 2024-06727-01).

### Data analysis

The analysis of the data was performed using qualitative content analysis inspired by Graneheim and Lundman [15–17].

All discussions were digitally recorded and transcribed verbatim. One of the researchers (AD) read the texts several times to get familiar with the data and then identified meaning units. The meaning units were condensed and thereafter abstracted into codes by AD. The co-authors went through the material of one focus group each to triangulate the identification of meaning units and the coding. Thereafter, all authors compared, sorted and resorted the codes. Sub-categories and categories were discussed, refined and defined in collaboration until consensus was reached by the authors. Finally, an underlying theme emerged representing the latent content of the text. Member checking was not conducted, as the data were highly homogeneous within the groups and no contradictory patterns emerged. This approach follows the model described by Graneheim and Lundman, where an overarching theme captures the underlying meaning of the data and brings together the different aspects of the manifest content [16]. Examples of meaning units, condensation, coding and categorisation are presented in Table 2. No qualitative analysis software was used; data were managed in Microsoft Word and analysed manually using printed text segments. The Consolidated criteria for reporting qualitative research (COREQ) checklist was used in the preparation of the manuscript (Supplementary material).

**Table 2.** Examples of text condensation and coding.

Meaning unit	Condensed meaning unit	Code	Subcategory	Category
‘I think the time factor is the main reason why you don’t do this with all patients, because… it takes time’ (Focus group 4)	The time factor is the main reason why you don’t do this with all patients	The time factor is crucial for the number of MRs	Leadership	Obstacles and facilitators
‘An underutilized tool or aid that… yeah, but I wish we could have done it more… but I’ve found it very useful the few times I’ve done it.’ (Focus group 1)	Underutilized tool/aid. Wish we could do it more, have been very useful	MR is an underutilized tool.	The doctor’s attitude	Obstacles and facilitators
‘…and there is also a medical safety aspect in that, to have the pharmacist’s eyes reviewing it…precisely from a medication perspective.’ (Focus group 4)	It is also a medical safety aspect with a review from pharmacological perspective	Pharmacological review provides medical safety	Patient benefit	Value-adding aspects
‘…so, it kind of becomes a wake-up call, it becomes like a learning experience.’ (Focus group 1)	MRs becomes like a wake-up call, like a learning.	MRs provides learning	Learning	Value-adding aspects

### Preconceptions

The research team consisted of both pharmacists (AD, CL) and general practitioners (SM, BBB), all female. AD, CL and SM have several years of experience in interprofessional MRs. All researchers have a special interest in patient safety work for elderly patients. SM and CL are actively involved in the area of medication safety for the elderly in the county. The moderator, AD, completed a course in qualitative methods for doctoral students prior to the interview process, and the rest of the research team has solid experience in qualitative methodology. Reflections on the preconceptions and how they might have influenced the data collection and analysis were carried out throughout the process.

## Results

From the meaning units, we identified four categories with 17 subcategories (Table 3). The four categories consisted of **obstacles and facilitators**, **interprofessional collaboration with the pharmacist**, **the physician’s responsibility** and **value-adding aspects**. A theme emerged from the underlying meaning of the categories: **A value-creating intervention with logistical barriers**. The categories are presented here with subcategories in italics.

**Table 3.** Subcategories, categories and theme.

Subcategory	Category	Theme
The physician’s knowledge of MRs The physician’s attitude Leadership Logistical issues Several separate care providers Pharmacist resource	Obstacles and facilitators	A value-creating intervention with logistical barriers
Different perspective Written recommendations Verbal communication Colleague	Interprofessional collaboration with the pharmacist	
Clinical judgement Measures and follow-up Possible planning for future medication reviews	The physician’s responsibility	
Patient benefit Value-creating in daily work Learning Cost effectiveness	Value-adding aspects	

Some elements, such as time, emerged as significant during the analysis. Although they were initially considered as potential subcategories, they were instead integrated into broader categories where they were more relevant in relation to the overall findings.

### Obstacles and facilitators

One of the identified subcategories concerns *the physician’s knowledge of MRs*. A fundamental presumption for initiating an MR is that the physician is aware of the possibility of accessing this service, which several participants were not, even though their local colleagues were using the method. Furthermore, knowledge of the workflow is necessary, which was often lacking, even among participants who were aware of the possibility of MRs.

‘Yeah, I don’t know what to do, it’s the first question. So practically, how do I get help, and like, when. The logistics around it is really my only hurdle’ (Focus group 3).

The participants considered that regular use of the method could ensure that the right patients in need of MRs are selected, and that the habit of performing MRs becomes established. As one participant expressed:

‘I think the threshold is before you’ve sort of started using it” (Focus group 3).

*The physician’s attitude* was illuminated in the discussions as significant for whether MRs were initiated and whether measures were implemented or not. An overwhelming majority expressed that MRs are important, instructive and underutilised. They considered it an excellent tool to increase the quality of medication treatment. However, the participants raised the point that some colleagues had no interest in MRs.

The participants stated that the *leadership* of the healthcare centre has a major impact on the work with MRs. The manager of the healthcare centre is a facilitator in ensuring that the importance of MRs for patient safety is understood. The manager also provides the conditions for implementation, which, for the physicians, mainly concerns getting enough time allocated. Difficulties with allocated time were seen as a potential barrier to performing more MRs.

‘You really need to have time set aside for this [MRs] to lead to concrete actions and truly benefit the patients’ (Focus group 4).

Clear financial incentives from the county were thought to influence the managers’ attitude. Some of the participants expressed a desire for more information to be provided to the managers regarding the benefits of MRs for patients and society.

A major part of the discussions concerned *logistical issues*. A simple way to initiate an MR was demanded.

‘I would really just like to be able to just press a button and then this will be set in motion and gets done’(Focus group 3).

The existence of local routines was perceived as a factor for success. The preparatory work was deemed to be preferably handled by other healthcare staff in order to save the physician’s time for the parts that must be the physician’s responsibility. Some of the participants stated that support from municipal care for both initiation and symptom assessment (PHASE-20) could make the work easier. However, sometimes a lack of cooperation between primary care and home healthcare posed an obstacle. An automatic or structured approach in selecting patients for MRs based on specific criteria, such as polypharmacy or repeated hospitalisation, was seen as potentially facilitating. Some participants expressed a desire to schedule MRs before the annual check-up to facilitate the workflow.

An obstacle to MRs, pointed out by the participants, was when *several separate care providers* were involved regarding the same patient. This was due to concerns regarding different areas of responsibility and hesitation to alter a specialist’s prescriptions.

Within the discussed model of MRs, a pharmacist identifies potential drug-related problems and provides recommendations to the physician on how to solve the problem. Concerns about limited access to *pharmacist resources* emerged as a potential barrier to MRs. Therefore, it was considered particularly important that the pharmacist’s work is not done unnecessarily but is genuinely followed up by the physician.

### Interprofessional collaboration with the pharmacist

The high level of competence among the pharmacists regarding pharmacology was very much appreciated among the participants.

‘…you’re surprised by their competence and thoughts, and their knowledge about both medications and medical indications and…’ (Focus group 4).

The participants also discussed the experience of the pharmacist, contributing a *different perspective* compared to that of the doctors, which was seen as positive. Similarly, the pharmacists’ thorough preparatory work in reviewing medical records was appreciated and described as time-consuming detective work.

‘It is almost never possible to read out when it was started [*the medication*], when you intend to end [*the medication*], and so on, so it requires… Sometimes you have to search through a thousand different medical records and note ‘yes, but here it seems to have started, and here is a pause’. And it is very time-consuming. You feel more like Sherlock Holmes than a doctor’ (Focus group 4).

The pharmacists’ preparatory work was seen as enabling the identification of issues such as unclear indications, the need for effect evaluation and loose ends.

The *written recommendations* from the pharmacists were brought to attention. Several of the participants considered them as clear, structured and well-formulated with good scope. They were also perceived as sensitive and without pointers, which was seen as positive for both physician and patient. Some of the participants pointed out that the recommendations were not given in imperative form but rather as ‘consider this’ instead of ‘do this’.

‘I thought that they had actually even thought about it and delicately chosen certain expressions, ‘can be thought of’, and were not imperative, but more like ‘this could exist, you have to think about it if you’… like a service and not that you are trying to sort of control’ (Focus group 3).

The participants reflected that *verbal communication* with the pharmacist provides more value but is also more time-consuming. Some of the participants felt that the written recommendation is sufficient, with the option to add contact when needed. Others experienced that verbal communication contributes to more measures being implemented. The benefit of increased learning through discussing possible actions in a group was underlined. However, it was noted that some found it difficult to arrange an appointment with the pharmacist.

In addition to participation in MRs, the pharmacist’s knowledge was perceived as valuable and added benefits as a *colleague*. The participants saw opportunities in receiving help from a pharmacist as a discussion partner on pharmacological issues, such as interactions, and concerning the situation with medication shortages. A request for help with medication reconciliation before patient visits and when renewing prescriptions was also expressed.

‘We’ve wanted for a really long time… I mean, my dream is to have a pharmacist at the health centre who could handle all… basically all prescription renewals, and from that aspect go through with the patient like ‘yeah, but how do you use this, how often do you take it’, and like go through the medication list and have a regular… being a colleague in the daily work on site would have been fantastic’ (Focus group 4).

### The physician’s responsibility

The sub-category *clinical judgement* emerged from the participants’ perception that the physician is responsible for decisions regarding possible changes in therapy, and that these decisions must be based on clinical knowledge of the patient. Some perceived the order of the suggested measures from the pharmacist as ranked by clinical relevance, but it was again emphasised that this prioritisation should be done by the clinician.

‘You have to start with what is relevant and what is sustainable. I mean, the one with Metformin and creatinine, that’s obvious, it’s important. But to stop eye drops or, you know, those that are less… or B12 that the patient has been taking for 10 years, which discussion is important to bring up now.’ (Focus group 3).

A concern was raised that suggestions for medication adjustments in relation to symptoms might lead to neglecting the investigation of somatic symptoms/problems, which is why follow-up was considered important. Furthermore, some participants described it as sometimes overwhelming to obtain information about all the patient’s symptoms from the symptom assessment scale, used within the MR.

The participants agreed that *measures and follow-up* after an MR can be time-consuming. Allocated time was stated to be a prerequisite for taking measures, including adequate follow-up. Regarding patients with municipal care or with patient-specific dispensed medications, adjustments in medication and evaluations were considered easier to implement. Patient-specific dispensed medications refer to the repackaging of solid oral medications used regularly into unit-dose bags for each time of administration. For patients who manage their medication themselves, a need for follow-up with the patient through telephone calls or visits was discussed.

‘Then it requires quite a lot of time when it comes to follow-up, because you have to sit and call all these patients.’(Focus group 2).

Concerns about making too many adjustments at once among these patients were also mentioned. The participants described that they sometimes have difficulty motivating patients regarding medication adjustments, and that relatives occasionally also may have strong opinions.

Concerning *possible planning for future medication reviews,* several of the participants expressed that they would appreciate the ability to select and schedule MRs for appropriate patients, for example, before annual visits, although a few found it difficult.

‘It would have been nice to initiate this the month before the annual check-up or so, so you have plenty of time during the check-up to actually incorporate all these recommendations.’ (Focus group 2).

### Value-adding aspects

The participants clearly expressed that MRs were perceived as important for the patient group and that they added *patient benefit*. They stated that MRs provide medical security, create safety for the patient and reduce the risk of errors. Some of the participants stated that MRs could provide added value for patients living independently, as these patients may sometimes be a bit forgotten. They also clarified that the method could entail that the pharmacists identify loose ends that require follow-up, and that MRs are a valuable service to offer patients.

‘It is valuable for the patients and then somehow it also makes it easier for us. If the patients receive more appropriate medication treatment so… the care improves, and the patient feels better and maybe requires fewer visits’ (Focus group 1).

The participants stated that MRs facilitate their processes and are *value-creating in daily work*. It was described as a luxury to receive structured support that can make day-to-day tasks more efficient. Issues brought to light that would otherwise go unnoticed.

‘…I also think it’s great, and it’s one of the few things, new things, that actually makes work easier. Or new and new, but one of…an additional thing that makes the job easier… Almost always it’s just more work and more work and more work, but this makes it faster I think’ (Focus group 1).

Many found it positive to be reminded even of obvious things. Some of the participants mentioned that the written recommendations from the pharmacist in the MR can give support in the interaction with the patient. The symptom assessment scale, PHASE-20, was perceived as a good instrument, both for identifying symptoms and as an educational tool, together with the patient.

‘Again, you can use it pedagogically back to the patient as well, that ‘now we have done this and you have filled in your symptoms, and then we have the expert on medications pointing out that this medicine can contribute to you feeling tired or a bit forgetful or…’ And then you have some additional backup to get the patient on board’ (Focus group 4).

On the other hand, some of the participants had a concern about the risk of identifying symptoms that patients might otherwise not have sought help for. Still, others believed that MRs could result in a slightly reduced need for healthcare visits.

An issue discussed by the participants was the need for increased knowledge of pharmacology and medications among physicians. MRs provide increased knowledge and were appreciated as a form of professional competence development. Some participants wanted MRs to become a mandatory part for residential physicians. Several participants emphasised that MRs provide *learning* and a mindset that spills over to benefit other patients.

‘So, I believe there is a great pedagogical point in that, so to speak. We can’t do a medication review for everyone, but one might be made aware of an interaction or an inappropriate combination which can be taken to others. Because it does spread. Even if it might be a bit slow, you do get a different mindset, so I absolutely think it’s good’ (Focus group 2).

Group discussions regarding MRs were considered as educational for all participants, and in addition, enjoyable and stimulating sessions that contribute to increased competence beyond one’s own patients.

The issue of *cost effectiveness* was raised, and also that drug-related problems cause costs. The participants believed that MRs could provide cost savings through reduced healthcare consumption, but also through lower medication costs.

‘…that we do it in the right way, then it’s beneficial for our patients, but it is also beneficial for healthcare and society in general.’ (Focus group 4)

## Discussion

### Main findings

This qualitative study aimed to explore primary care physicians’ views on MRs for patients who live independently. The participants expressed an overwhelmingly positive attitude towards the benefits of the method, although logistical barriers were also identified as obstacles to its implementation. Factors influencing the occurrence or absence of the process of an MR were highlighted, leading to the identification of the category obstacles and facilitators. The participants expressed positive views regarding their interprofessional collaboration with the pharmacist during the process, even though their preferences regarding written versus verbal communication with the pharmacist varied. The significance of the physician’s responsibility in the MR was emphasised, along with the critical qualities that contribute to value-adding aspects for both doctor, patient and society.

The latent theme of a value-creating intervention with logistical barriers captures the perceived challenges and contradictions associated with the appreciated method and its further implementation.

### Comparison with existing literature

In this study, limited knowledge of the possibility and process of MRs for patients living independently was noted among the participants. Challenges in information, dissemination and implementation of new work methods are well known and can be addressed in various ways. Similar challenges regarding MRs have been described by Dhillon et al. and Duncan et al., such as unclear routines and time constraints, which underline the need for structured support and well-defined workflows [13,14]. A review from 2023 concluded that MRs are more successful through collaboration in well-integrated multidisciplinary teams [3]. Having potentially closer collaboration with the clinical pharmacists would increase the knowledge base and thus facilitate the process. In addition, having a pharmacist on site at the local health care centre could potentially be one way to provide more thorough information regarding MRs. That might also entail opportunities for additional support, such as assistance in pharmacological issues, medication reconciliations, or prescription renewals, as mentioned by the participants. Gaël et al. pointed out enhanced collaboration with pharmacists as a possible support for physicians in Belgium to better handle the complex management of prescribing for older patients [18]. In line with our results, Duncan et al. emphasise the importance of a good working relationship between physicians and pharmacists and highlight the potential of pharmacists’ supportive role in alleviating the physicians’ workload [14]. This also includes the recognition of the need for clearly defined roles in collaborative work in the UK primary care. In line with these findings, the participants in our study appreciated the pharmacists’ non-imperative and well-structured recommendations, which supported physician autonomy and contributed to a respectful working relationship. These similarities in perceptions were observed despite differences in healthcare settings and organisational structures.

The participants in our study clearly considered MRs to be beneficial for the patients, stating that MRs provided learning and created a cascading impact. Findings from an Australian review also indicated value for patients by improving medication safety through resolving medication-related problems through collaboration between general practitioners and pharmacists [19]. Previous studies have shown there are fewer hospitalisations following interprofessional MRs [20,21].

According to the Swedish National Board of Health and Welfare, a physician should be responsible for MRs [5]. As underlined by the participants in our study, it is of great importance that the physicians make their own clinical judgement regarding which measures are relevant to be taken within the MR. With information provided by the pharmacist, the physician could make more well-informed decisions, while considering the clinical knowledge of the patient’s individual circumstances. The physician is responsible for the treatment and for systematic follow-ups and must adapt the medical therapy based on the patient’s unique conditions. This contrasts with the U.S. model, where pharmacists may initiate or modify treatments under Collaborative Practice Agreements [4]. In our study, the participants emphasised the importance of retaining clinical responsibility and making individualised decisions based on their knowledge of the patient. By systematically utilising MRs, the physician could ensure patient safety and, through learning, disseminate the knowledge to benefit other patients as well.

The participants in our study exhibited an almost unanimous positive perspective on MRs for patients living independently, aside from logistical issues. This contrasts, to some extent, with another Swedish study from 2018, where a slightly more negative attitude towards MRs was reported [22]. However, differences in setting and methodology should be noted. In their context, no pharmacist took part in the MRs; the physician and nurses did all the work themselves, which may have influenced the views. A feeling of being forced to perform MRs according to guidelines was also noted in that study, and the authors concluded that physicians and nurses should take part in the construction of guidelines to enhance applicability. The involvement of healthcare staff in constructing local routines for MRs could advantageously be applied in our setting as well, given the logistical barriers identified. Individuals locally situated with a particular interest in the issue could facilitate the process. Depending on how the role is structured, this could also entail learning opportunities for other professions besides physicians. Previous studies have enlightened the mutual learning opportunities arising from the collaboration between professions in an MR [23].

The participants pointed out some significant differences regarding MRs for patients living independently, depending on whether they received support from municipal care or not. Similar to Carlqvist et al. 2024, we found that the importance of prioritising patients living independently was underscored [24], with the motivation that they often handle their own medications and do not receive as much attention as patients at nursing homes. This is reinforced by the fact that, in a previous study, we found that the vast majority of this patient group were exposed to at least one drug-related problem, with an average of 3.9 drug-related problems per patient [11]. In today’s healthcare context, characterised by numerous guidelines and care programmes, an MR may contribute to the ability to provide personalised care and individualise medication treatment. Since the patients living independently are often more involved in their medication treatment, the participants also regarded the MRs as a valuable service to offer their patients. Despite the many advantages, both logistical issues and the follow-up procedure were considered to be more challenging and time-consuming compared to MRs at nursing homes. In general, the problem of setting aside time is pointed out repeatedly, along with other practical obstacles.

Our findings are consistent with those of Dhillon et al., who reported that Australian general practitioners valued pharmacist input but faced workflow challenges [13]. Similarly, our participants described logistical barriers – such as limited time and lack of clear routines – as key obstacles to implementing MRs, despite their positive attitudes towards the method. The identified obstacles to conducting more MRs emphasises the importance of continuously developing efficient work methods and tailoring solutions that are integrated into the organisation. However, the participants also noted that the MRs could enhance their efficiency, and thereby potentially facilitate more time for, among other things, follow-ups.

Although not all patients can receive an interprofessional MR due to limited resources, the process itself was described as educational and helpful in changing how physicians think about medication-related issues. As one participant expressed, ‘you do get a different mindset,’ referring to how MRs raise awareness beyond the individual patient. By incorporating MRs more routinely into clinical practice, physicians may develop a broader approach to pharmacological risks and opportunities, which could benefit other patients indirectly. This broadened perspective might also influence prescribing patterns among physicians.

## Findings in a theoretical framework

To provide an additional perspective on the findings of this study, the process of implementation of MRs can be interpreted using Lewin’s Change Management Model [25]. Developed by Kurt Lewin in the 1950s, this is one of the models that has been applied to guide change processes in healthcare [26]. The model describes three stages of change: unfreezing, changing, and refreezing. This framework can be used to understand the change process, analyse responses to change and facilitate the transition from one state to another. In our focus groups, it was evident that some primary healthcare centres and/or physicians remained in step one of Lewin’s model, unfreezing, where existing norms and habits need to be reconsidered to enable change. The outcomes from this study could serve as an important support to identify and understand obstacles in the current situation and to accentuate the positive driving forces. A major part of the physicians and healthcare centres are considered to be in the second step, undergoing change. The new process and method have been introduced; thus, information and support were considered crucial for individuals to embrace them. Measures must be taken to overcome identified obstacles. As stated by the participants, leadership plays an important role in guiding and encouraging employees during this phase. What remains after the method has been fully implemented is to refreeze the new status, to keep the value-creating aspects, as described by the participants. This allows MRs for patients living independently to become a natural part of routine and culture in primary care. The refreezing phase represents the third and last step in Lewin’s model. The participants in the fourth focus group, who had more extensive experience, seem to be more devoted to the method and appear to be closer to evolving to the third refreezing step. Understanding where different individuals are within Lewin’s model may help to develop strategies to support the long-term implementation and integration of MRs for patients living independently in primary care.

## Method discussion

This is, to our knowledge, the first Swedish study assessing primary care physicians’ views on interprofessional MRs for patients living independently.

The strategic selection of participants, including both physicians with and without prior experience of MRs for patients living independently, may be considered a limitation, as some participants may have shared perceptions rather than direct experiences. However, we consider this diversity a strength, as it allows us to explore not only experiences of participating in MRs, but also reasons for non-participation, perceived barriers and attitudes toward the method. All participants worked in the same primary care context and had been offered the opportunity to engage in MRs, which provided a shared frame of reference. The mix of participants contributes to yielding richer content and enables a more comprehensive identification of obstacles, such as practical or informational. The choice of focus group discussions is appropriate since discussions among the participants could generate more aspects and ideas than might have been possible to capture in individual interviews. The result could support the process of developing the model for MRs, identifying barriers and how to overcome them. A potential risk with the method is that individual participants may dominate the discussions, but this was counteracted in this study by the moderators ensuring balanced speaking time and by using the semi-structured interview guide.

The combination of different professions in the research team could contribute to diverse perspectives in the interpretation of the results. The triangulation performed throughout the analysis and writing process strengthens the internal validity. The authors’ pre-existing understanding and particular interest in the topic may have influenced the evaluation of the collected material, but could also be viewed as an asset, thus adding a deeper understanding and facilitating more fluid discussions, potentially resulting in richer material when attending to reflexivity. Nevertheless, it cannot be excluded that preconceptions may have influenced the data collection and interpretation. Besides this, the fact that AD is a pharmacist who works with MRs might have constrained participants’ criticism of the pharmacists’ role. However, in all focus group discussions, a physician served as a co-moderator, thus providing an additional perspective.

One of the focus group discussions was held as a digital meeting. A concern was that this might inhibit a fluid discussion. On the other hand, the participants were all experienced physicians with confidence in their role in working with MRs, which counteracted the risk. In addition, the discussion turned out to provide rich content. Moreover, the use of a digital meeting enabled the inclusion of physicians from different geographic areas in the same group.

This study took place in Skåne County in southern Sweden, where a specific model for MRs is utilised. Given this, the results would likely be affected if transferred to another region or country with different settings. Despite this potential limitation, there are examples of studies (Refs. [13,23]) with comparable settings and results, which strengthen transferability.

## Future research

An important topic for future research is to assess the patient’s perspective on MRs and on medication adjustments. An MR often results in adjustments to the patient’s medication treatment, and the patient’s attitude towards these changes is crucial for successful implementation. The patient’s perspective would provide the opportunity to adapt the model and thus maintain the highest possible level of patient safety.

It could also be valuable to explore how pharmacists and, when relevant, nurses view the MRs. They both play important roles in making medication use safer [27,28], and their perspectives on the model could support further development.

## Concluding statement

This study provides insights into how primary care physicians perceive and experience MRs for independently living patients. The overarching theme, *a value-creating intervention with logistical barriers,* captures the dual nature of the findings: MRs are seen as highly beneficial for patient safety, clinical decision-making, and professional learning, yet their implementation is hindered by practical challenges such as time constraints and unclear routines.

The findings reflect a broader tension between clinical priorities, limited resources, and patient safety goals. Addressing these challenges is essential to strengthening physicians’ ability to integrate MRs into patient-centred care. With targeted structural support, MRs could evolve from being valued but sporadic into a routine part of clinical practice, enabling more systematic identification of medication-related risks and more personalised treatment. In the long term, this may help avoid iatrogenic harm and unnecessary healthcare use.

## Supplementary Material

Appendix 3_COREQ_Checklist_LMG OBO_oktober.pdf

Appendix 2_Information to participants.docx

Appendix 1 Semi structured interview guide.docx

## Appendices

1. Semi-structured interview guide
2. Information to participants
3. The Consolidated criteria for reporting qualitative research (COREQ) checklist

## Appendix A – interview guide

### Semi-structured interview guide interprofessional MRs, independent living

**Has anyone participated in MRs (for independently living patient)? Please tell us what you think about it and how you experienced it.**

- Patient benefit? Quality improvement? Negative aspects? Logistics?

**Checklist (=has this been addressed?):**

1. Potential improvement
  - Why do you think so few MRs are performed for these patients?
  - Possible barriers?
  - How could the work be made easier?
  - What is already working well?
2. Routine for work
  - What are your thoughts about this? Experiences of the different variants?
  - What about allocated time, preparation, patient selection etc?
3. The journal entry: Tell what you think about the pharmacist’s journal entry and how you use it.
  - Clear enough? Amount of information? (how do needs vary depending on whether there is an additional verbal discussion or not?)
4. Feedback (between pharmacist and physician)
  - Experiences
  - Advantages of written versus written and additional verbal discussion.
5. Continued work with MRs
  - What is your view on potential continued work with MRs for independently living patients? (Increased/decreased extent? In what way should it continue?)
6. There are MRs where ‘everything’ is available (symptom assessment completed, journal entry from pharmacist), but there is no note that anyone has taken it into account – what do you think could be the cause?
  - Reasons?
  - How to avoid?
7. If participated i MRs: why are some of the proposed measures taken into account but not all (and sometimes none)?
  - The patient? Lack of time? Difficult to implement? Etc.
  - How do you organise the work on the proposed measures? Any specific structure/plan?
8. How do you view MRs as skills development?
9. Is there any additional support you would want to receive from a pharmacist in your work with this patient group?

#### Summary

1. What is the most important to highlight from what was said?
2. Summary
3. Have we missed covering anything?

## Appendix B – Information provided prior to the focus group sessions

Hello!

The scheduled focus group session at your location is coming up soon, on [date] at [time]. [Name] and I will be attending, and we will arrive a little in advance to ensure we can start on time. Below you will find information we kindly ask you to read before the session.

### Purpose

The purpose of the focus group is to capture thoughts and perceptions about, as well as any experiences of, interprofessional medication reviews for patients living independently. This may include both opportunities and challenges. There are no right or wrong answers, the aim is simply to gather your views and reflections.

### Ahead of the group discussion, you are welcome to reflect on the following questions

- What are your thoughts on medication reviews for independently living patients (including both those with and without municipal care)?
- What opportunities and challenges do you see in conducting medication reviews for this patient group?

### Procedure during the session – discussion guidelines

Certain topics and questions will be raised during the session. You will have space to discuss these and to contribute anything else you wish to share within the topic area. The conversation will be recorded, but all data will be anonymised during processing. The recording is made to ensure that no important information is overlooked and to capture all aspects of the discussion.

- We have approximately 50–60 minutes available.
- If possible, please set your mobile phone to silent. If you need to take a call, kindly step outside and return as soon as you are finished.
- It is important to highlight everyone’s views. Please be honest and share your thoughts, there are no right or wrong answers.
- All thoughts and comments are valuable. You are welcome to respond to each other’s contributions.
- Everything shared in the group stays in the group – the discussion should not be shared outside the session.

### Consent

During the focus group session, all participants will also receive verbal information and have the opportunity to ask questions. If consent to participate in the research project is given, participants will be asked to sign a consent form. If there are any questions prior to the session, feel free to get in touch with me.

Kind regards

[Name]
