# Supplementary material for: ‘You get a different mindset’ – primary care physicians’ perceptions of interprofessional medication reviews for patients living independently
Source: Scand J Prim Health Care. 2026 Jan 2;44(1):2604036. doi: 10.1080/02813432.2025.2604036 (PMC12777794; doi:10.1080/02813432.2025.2604036)
Supplement: Appendix 2_Information to participants.docx [file IPRI_A_2604036_SM4722.docx]

***Appendix B – Information provided prior to the focus group sessions:***

Hello!

The scheduled focus group session at your location is coming up soon, on [date] at [time]. [Name] and I will be attending, and we will arrive a little in advance to ensure we can start on time. Below you will find information we kindly ask you to read before the session.

**Purpose**

The purpose of the focus group is to capture thoughts and perceptions about, as well as any experiences of, interprofessional medication reviews for patients living independently. This may include both opportunities and challenges. There are no right or wrong answers, the aim is simply to gather your views and reflections.

**Ahead of the group discussion, you are welcome to reflect on the following questions:**

- What are your thoughts on medication reviews for independently living patients (including both those with and without municipal care)?
- What opportunities and challenges do you see in conducting medication reviews for this patient group?

**Procedure during the session – discussion guidelines**

Certain topics and questions will be raised during the session. You will have space to discuss these and to contribute anything else you wish to share within the topic area. The conversation will be recorded, but all data will be anonymised during processing. The recording is made to ensure that no important information is overlooked and to capture all aspects of the discussion.

- We have approximately 50-60 minutes available.
- If possible, please set your mobile phone to silent. If you need to take a call, kindly step outside and return as soon as you are finished.
- It is important to highlight everyone’s views. Please be honest and share your thoughts, there are no right or wrong answers.
- All thoughts and comments are valuable. You are welcome to respond to each other’s contributions.
- Everything shared in the group stays in the group – the discussion should not be shared outside the session.

**Consent**

During the focus group session, all participants will also receive verbal information and have the opportunity to ask questions. If consent to participate in the research project is given, participants will be asked to sign a consent form. If there are any questions prior to the session, feel free to get in touch with me.

Kind regards

[Name]
