# Supplementary material for: ‘You get a different mindset’ – primary care physicians’ perceptions of interprofessional medication reviews for patients living independently
Source: Scand J Prim Health Care. 2026 Jan 2;44(1):2604036. doi: 10.1080/02813432.2025.2604036 (PMC12777794; doi:10.1080/02813432.2025.2604036)
Supplement: Appendix 1 Semi structured interview guide.docx [file IPRI_A_2604036_SM4721.docx]

**Appendix 1 – interview guide**

**Semi-structured interview guide interprofessional MRs, independent living**

**Has anyone participated in MRs (for independently living patient)? Please tell us what you think about it and how you experienced it.**

- Patient benefit? Quality improvement? Negative aspects? Logistics?

**Checklist (=has this been addressed?):**

1. Potential improvement

- Why do you think so few MRs are performed for these patients?
- Possible barriers?
- How could the work be made easier?
- What is already working well?

2. Routine for work

- What are your thoughts about this? Experiences of the different variants?
- What about allocated time, preparation, patient selection etc?

4. The journal entry: Tell what you think about the pharmacist’s journal entry and how you use it.

- Clear enough? Amount of information? (how do needs vary depending on whether there is an additional verbal discussion or not?)

3. Feedback (between pharmacist and physician)

- Experiences
- Advantages of written versus written and additional verbal discussion.

5. Continued work with MRs

- What is your view on potential continued work with MRs for independently living patients? (Increased/decreased extent? In what way should it continue?)

6. There are MRs where ”everything” is available (symptom assessment completed, journal entry from pharmacist), but there is no note that anyone has taken it into account – what do you think could be the cause?

- Reasons?
- How to avoid?

7. If participated i MRs: why are some of the proposed measures taken into account but not all (and sometimes none)?

- The patient? Lack of time? Difficult to implement? Etc.
- How do you organise the work on the proposed measures? Any specific structure/plan?

8. How do you view MRs as skills development?

9. Is there any additional support you would want to receive from a pharmacist in your work with this patient group?

**Summary:**

1. What is the most important to highlight from what was said?
2. Summary
3. Have we missed covering anything?
